# Proper Selection Does Make the Difference: A Propensity-Matched Analysis of Percutaneous and Surgical Cut-Down Transfemoral TAVR

**DOI:** 10.3390/jcm10050909

**Published:** 2021-02-25

**Authors:** Marco Gennari, Marta Rigoni, Giorgio Mastroiacovo, Piero Trabattoni, Maurizio Roberto, Antonio L. Bartorelli, Franco Fabbiocchi, Gloria Tamborini, Manuela Muratori, Laura Fusini, Mauro Pepi, Paola Muti, Gianluca Polvani, Marco Agrifoglio

**Affiliations:** 1 Department of Cardiovascular Surgery, IRCCS Centro Cardiologico Monzino, 20100 Milan, Italy; gio.mastroiacovo@hotmail.it (G.M.); piero.trabattoni@ccfm.it (P.T.); maurizio.roberto@ccfm.it (M.R.); marco.agrifoglio@ccfm.it (M.A.); 2 Department of Industrial Engineering, University of Trento, 38100 Trento, Italy; marta.rigoni@unitnt.it; 3 Department of Oncology and Health, Evidence, and Impact, McMaster University, Hamilton, ON L8S 4L8, Canada; Paola.Muti@unimi.it; 4 Department of Biomedical and Clinical Sciences “Luigi Sacco”, University of Milan, 20100 Milan, Italy; antonio.bartorelli@ccfm.it; 5 Department of Invasive Cardiology, IRCCS Centro Cardiologico Monzino, 20100 Milan, Italy; franco.fabbiocchi@ccfm.it; 6 Department of Cardiovascular Imaging, IRCCS Centro Cardiologico Monzino, 20100 Milan, Italy; gloria.tamborini@ccfm.it (G.T.); manuela.muratori@ccfm.it (M.M.); laura.fusini@ccfm.it (L.F.); 7 Clinical Area Director, IRCCS Centro Cardiologico Monzino, 20100 Milan, Italy; mauro.pepi@ccfm.it; 8 Department of Biomedical, Surgical and Dental Sciences, University of Milan, 20100 Milan, Italy; gianluca.polvani@ccfm.it; 9 Chief of Cardiovascular Surgery Department, IRCCS Centro Cardiologico Monzino, 20100 Milan, Italy

**Keywords:** TAVR, percutaneous access, vascular complications, surgical cut-down, transfemoral approach

## Abstract

Background. Transcatheter aortic valve replacement (TAVR) is an established technique to treat severe symptomatic aortic stenosis patients with a wide range of surgical risk. Currently, the common femoral artery is the first choice as the main access route for the procedure. The objective of this observational study is to report our experience on percutaneous and surgical cut-down transfemoral TAVRs comparing the two approaches. Methods. From January 2014 to January 2019, five hundred eleven consecutive patients underwent TAVR for severe symptomatic aortic stenosis. We analyzed only elective transfemoral procedures. After propensity score-matching based on age, sex, EuroSCORE II, mean aortic gradient, and left ventricular ejection fraction, we obtained two homogeneous populations: surgical cut-down (*n* = 119) and percutaneous (*n* = 225), which were labeled Group 1 and Group 2, respectively. Results. The main findings were that there were no significant procedural outcome differences between the two groups, but Group 2 patients had a shorter length of hospital stay and were more frequently discharged home. At follow-up, Group 1 patients had lower survival rates. Conclusions. An accurate preoperative assessment of the femoral access is mandatory to achieve satisfactory outcomes with transfemoral TAVRs. Nevertheless, the percutaneous approach allows shorter in-hospital stay and the need for rehabilitation, thus potentially decreasing the costs of the procedure.

## 1. Background

Transcatheter aortic valve replacement (TAVR) is an established procedure to treat patients with severe symptomatic aortic stenosis (AS) at high and intermediate surgical risk. After PARTNER 3 and Evolut R low-risk trials [1,2] showing non-inferiority to surgical aortic valve replacement (SAVR) of the latest generation balloon-expandable and self-expanding valves, it is expected for there to be an increase of the number of transcatheter-based replacements of the aortic valve.

**Figure 1:**
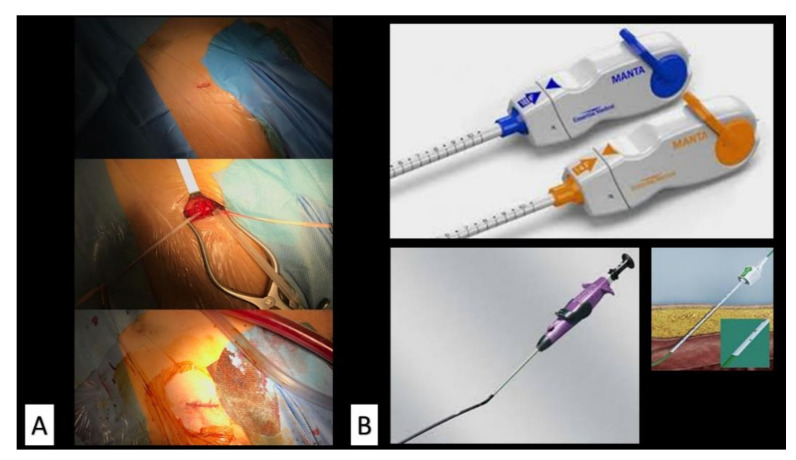
(**A**) Minimal surgical incision at the groin for the isolation of the common femoral artery. (**B**) Currently the most widely used vascular closure devices for large-bore arterial holes.

Currently, the transfemoral route is the preferred main access for the procedure [3] gained by either surgical cut-down or the percutaneous approach (Figure 1), the latter being the first choice whenever feasible. Despite the lower profile of the delivery catheters of the latest generation of transcatheter aortic valves (TAVs) and that the improvements of the performance of large-bore vascular closure devices (VCDs) had turned into an overall reduction in bleedings or major vascular complications, troubles at the vascular access site still have an impact on the outcome [4].

Surgical cut-down of the common femoral artery may allow better control and repair in case of complications, but it is burdened by all the classical surgical access-related problems [5] such as invasiveness, longer recovery, infection risk, lymphatic or neurological issues, and currently, a fully percutaneous approach with VCDs use is the preferred choice [6].

The aim of this observational study is to report our experience, outcomes, and follow-up of the transfemoral TAVRs with the currently available devices performed by either a surgical cut-down or a percutaneous approach.

## 2. Methods

**Figure 2:**
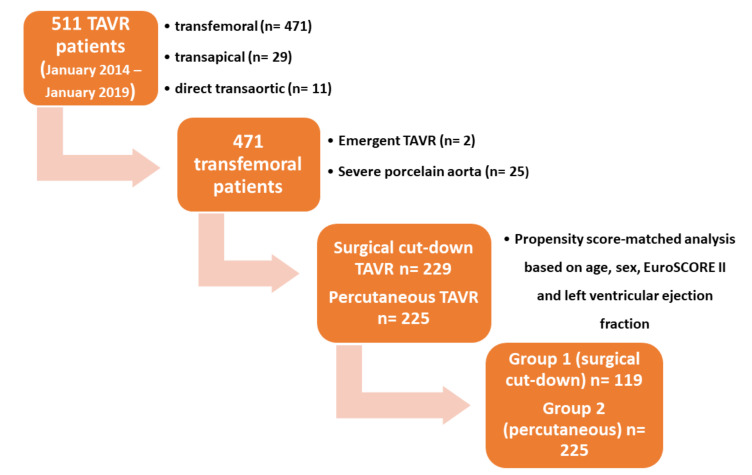
Flow chart of the study. Legend. TAVR = trancatheter aortic valve replacement.

This is an observational study of the perioperative and follow-up outcomes of both surgical cut-down and percutaneous transfemoral TAVRs, compared by a propensity score-matching of the two populations. Figure 2 depicts the patients’ selection process flow chart.

### 2.1. Patients

From January 2014 to January 2019, five hundred-eleven patients with severe symptomatic aortic stenosis were treated at our institution with both transfemoral balloon-expandable and self-expanding TAVR by the same surgical group. The decision to perform the transcatheter procedure was made by the local Heart Team according to established criteria [7]. The routes of delivery of the transcatheter heart valves (THVs) were femoral (*n* = 471), left ventricular apex (*n* = 29), and direct aortic (*n* = 11). Only elective transfemoral TAVRs have been analyzed in this work. Emergency procedures (*n* = 2) and patients with challenging porcelain aorta (*n* = 25) were excluded from the analysis.

We divided the remaining 444 patients into two groups—according to the surgical (*n* = 219) or percutaneous access (*n* = 225) to the femoral artery. Since these raw populations presented a relevant mismatch of the baseline characteristics (Table 1), we performed a propensity score-matched analysis based on age, sex, EuroSCORE II, body mass index (BMI), hypertension, diabetes, mean aortic gradient, and left ventricular rejection fraction, obtaining two homogeneous populations of 119 and 225 patients for the surgical and percutaneous group that we labeled Group 1 and Group 2, respectively. The majority of the patients were at intermediate surgical risk (Group 1 presenting a median EuroSCORE II of 4.09 and Group 2 presenting a score 3.77). The frailty burden was considered comparable for both populations after Heart Team evaluation. The decision to perform a surgical or a percutaneous approach was made in the Heart Team context after careful analysis of a contrast-enhanced non-electrocardiogram-guided multi-slice computed tomography (MSCT) of the abdominal aorta and femoral vessels. Briefly, in case of moderate, non-anterior wall calcifications and in the presence of adequate arterial diameters, the percutaneous approach was preferred. In case of borderline diameter with a sheath-to-femoral artery ratio (STFR) > 1.05 [8] or severe concentric calcifications and tortuosity, a direct surgical cut-down of the vessel was favored. Anyway, a final decision on the type of the femoral access was left to the discretion of the operator.

### 2.2. Ethical Committee

This work is based on a retrospective review of data prospectively collected with follow-up information retrieved by telephone calls and hospital records. This research has been approved by the Institutional Review Board (IRB), in accordance with the principles of the Declaration of Helsinki. The local ethical committee waived the requirement for individual consent for the study due to the retrospective nature of our analysis.

### 2.3. Surgical Cut-Down Technique

A 3-cm long transversal incision is made ≈1 cm above the inguinal fold. After that, the subcutaneous tissue is longitudinally dissected, and the common femoral artery is approached laterally to decrease the hazard of lymphatic injury. Once the proximal and distal segments of the artery are encircled with a vascular lace, the anterior wall is manually palpated to find the best area for the access. Hereby, a non-calcific area is chosen, a double (180° degrees apart) 5–0 purse-string proline stitch is placed, and a tourniquet snaring system is applied. Afterwards, the artery is directly punctured under vision and tactile feedback. At the end of the procedure, the sutures are tight and the pulse is evaluated; if a relevant stenosis is suspected, the artery is temporary clamped, the purse-string sutures are removed, and the arterial wall is repaired.

### 2.4. Percutaneous Access Technique

For all the percutaneous procedures, we utilize the double pre-closing technique with two 6Fr Proglide (Abbott, Chicago, IL, USA) deployed at the 10 and 2 o’clock positions. After removing the procedural sheath and tightening the sutures, a bleeding check is performed; if satisfactory hemostasis is achieved, the sutures are further bounded and then cut. If bleeding is still an issue, manual compression or contralateral crossover management is performed (with peripheral balloon occlusion and stent-graft placement when indicated). A similar management is adopted in case of significant femoral stenosis.

### 2.5. Outcomes and Follow-Up

Most of the patients were followed up in our outpatient clinic, and the follow-up was completed in 100% of them. About 85% were clinical follow-ups, while the remaining were made by phone calls. The outcomes analyzed were procedural results according to VARC-2 (Valve Academic Research Consortium-2) definitions [9], median length of hospital stay, discharge destination, mortality at follow-up, New York Heart Association (NYHA) class, and rehospitalizations. Major adverse cardiovascular and cerebrovascular events (MACCE) as well as survival data were collected.

### 2.6. Statistical Analysis

Descriptive variables were expressed by mean ± standard deviation (SD) in case of normal distribution, or by median and first and third quartiles (q1, q3 respectively) in case of non-normal distribution. The normality of the variables was tested with the Shapiro–Wilk test. The dichotomous variables or scores were expressed as frequencies and occurrence percentages.

Variables and outcomes were compared between the two groups using the most appropriate test according to the type and nature of the data among the t-test for independent samples, nonparametric Mann–Whitney U-test, Pearson’s chi-squared test, or Fisher’s exact test.

The Cox proportional hazard model was used to estimate the hazard ratio (HR) and 95% confidence Interval (95% CI) for all-cause mortality for the percutaneous group in respect to the surgical cut-down group. Moreover, Kaplan–Meier estimates analysis was used to generate a time-to-event curve for all-cause mortality, and all event mortality was stratified by access type (surgical cut-down or percutaneous).

All tests were 2-tailed, and a *p*-value < 0.05 was set for statistical significance.

Statistical analyses were performed using the Stata software (StataCorp LLC 1996-2021, 4905 Lakeway Drive, College Station, TX, USA).

## 3. Results

### 3.1. Baseline Characteristics

The baseline characteristics of the two matched groups are listed in Table 2. After the propensity-score matching, only the body mass index (BMI) differed between the two populations, being higher in Group 2 (*p* = 0.05). No differences were found in the incidence of previous coronary interventions (Table 3), either coronary artery bypass or percutaneous coronary interventions, while Group 1 presented a higher incidence of previous surgical aortic valve replacements (SAVRs) (*n* = 8, *p* < 0.01).

All the procedures (except one) were performed in deep sedation and oro-tracheal intubation. We analyzed only third-generation devices (Sapien 3, Edwards Lifesciences, Irvine, USA and Evolut R, Medtronic, Minneapolis, USA). Most of the procedures were accomplished using the balloon-expandable platform. No differences between the two groups were recorded on the diameter of the TAVs (Table 4).

Similarly, we did not observe any statistical differences in the femoral sheath diameter (14Fr and 16Fr Edwards eSheath or 14Fr EnVeo R InLine Medtronic sheath).

### 3.2. In-Hospital Outcomes

No relevant intraprocedural or periprocedural differences were found between the surgical and percutaneous groups in terms of MACCE, major bleedings, major vascular complications, or neurological complications according to VARC-2 criteria (Table 5).

### 3.3. Clinical Outcome at Follow-Up

The median follow-up for Group 1 was 949 days (interquartile range 624–1434) and for Group 2, it was 1039 days (interquartile range 703–1553, *p* = 0.27). The main differences are listed in Table 6.

The percutaneous group had a significantly shorter length of hospital stay and was more frequently discharged home, while the surgical group frequently needed a postoperative rehabilitation. We generally offer postoperative rehabilitation to the surgical patients because we want to follow the correct healing of the surgical access, while the percutaneous patients are candidates to rehabilitation only in case of specific situations, such as post procedural rhythm disturbances.

**Figure 3:**
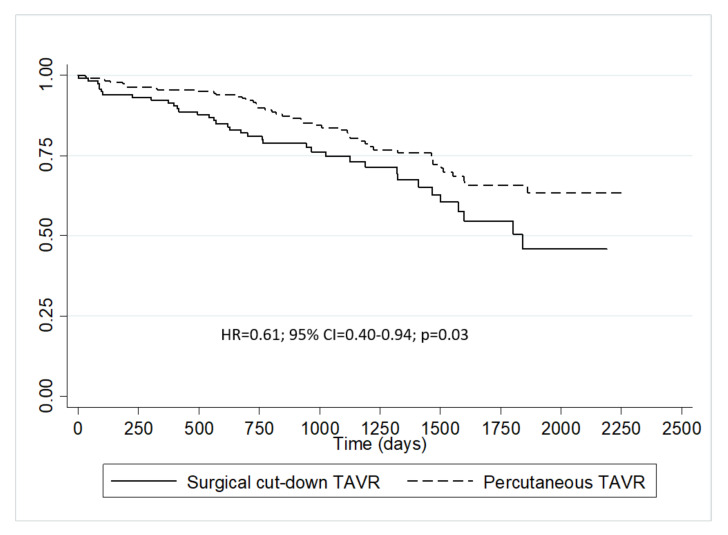
Kaplan–Meier survival estimates. Legend. CI= confidential interval; HR= hazard ratio; TAVR= transcatheter aortic valve replacement.

Kaplan–Meier survival estimates (Figure 3) showed a survival rate higher for Group 2, with a crude HR = 0.61; 95% CI = 0.40–0.94; *p* = 0.03. This means that in our analysis, the percutaneous approach was associated with a reduction in the morality hazard of 39% compared with the surgical counterpart.

## 4. Discussion

The main findings of this report are as follows: (i) an accurate preoperative assessment of the femoral vasculature is mandatory to achieve a low rate of major access-related complications; (ii) even though the operative results were similar for the two groups, patients underwent percutaneous access had a significantly lower length of hospital stay and were more frequently discharged home, potentially reducing the overall costs of the procedure; (iii) and finally, the percutaneous group presented higher survival rates at follow-up.

The latest generation of TAVs are deployed with a lower profile regarding their delivery catheters compared to the early generation devices [10,11]; this has led to a progressive shift toward a less invasive totally percutaneous approach to the femoral artery [12]. Although technical and expertise improvements in the last years have yielded to better outcomes, the incidence of vascular complications after TAVR is still reported to be between 8% and 30% [12].

Whatever the way of access, vascular complications after TAVR are linked to an excess mortality.

**Figure 4:**
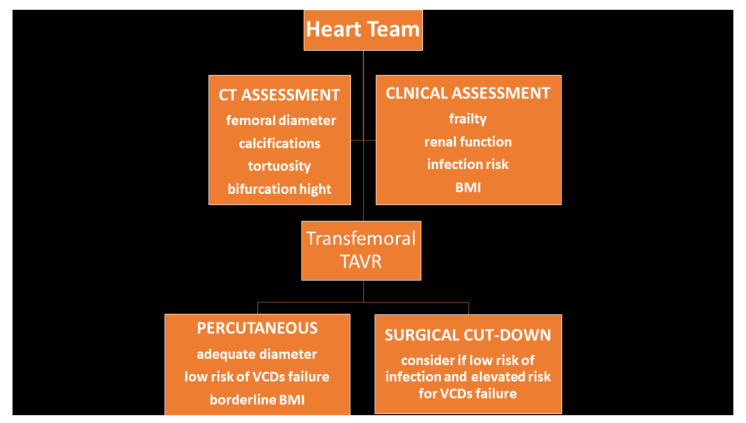
Flow chart of the decision-making to perform surgical cut-down versus percutaneous femoral access.

Accurate pre-procedural planning of the access is crucial for a safe vascular outcome. The MSCT is currently the main stem of the pre-TAVR assessment [13]; in particular, minimum diameters of the ilio-femoral axes, calcifications burden, and degrees of tortuosity are well-established features that may affect the risk of vascular injury. Although a profile reduction of the delivery systems and an ultrasound-guided approach to the femoral vessels [14] can reduce the hazard of vascular troubles, the choice of the right way of access still play a role. We report our Heart Team experience depicting comparable operative outcomes of both surgical and percutaneous approach, given a deep pre-procedural assessment of the clinical and anatomical characteristics (Figure 4).

Despite the favorable results, the greater invasiveness of the surgical cut-down of the femoral artery has led to an overall prolonged in-hospital stay and the need for postoperative rehabilitation.

This may affect the outcome in two ways. The slower recovery of the surgical group may predispose to prolonged immobilization and increased infections rates [15,16], which are all known concerns in these frail surgical candidates. Indeed, lymphoceles and paresthesia complications are quite more common when a surgical femoral isolation is performed [17]. We do not insert an inguinal drain on a regular basis, but only in selected populations such as obese patients or if an extensive arterial dissection is performed; in these cases, the drain can help reduce the common local post-procedural complications (such as infections, lymphoceles) at the expense of a prolonged in-hospital stay.

Secondly, the prolonged hospitalization may affect the costs of the procedure [18], despite the intrinsic costs of the percutaneous toolbox including the vascular closure devices (VCDs).

Most of the available data on the outcome of surgical or percutaneous transfemoral TAVR are derived from registries, case series, and observational studies, whose results are in line with our report [19].

Only a small, randomized clinical trial [20] has prospectively evaluated the outcomes between the two groups, determining that high-volume experienced centers may perform a total percutaneous approach with a low rate of vascular problems.

Finally, we report a higher mortality rate for the surgical group. Although definitive conclusions could not be driven from this evidence, a possible explanation is that the surgical group could have presented a higher global atherosclerotic cardiovascular burden (witnessed by eight valve-in-valve procedures), determining an increased mortality at follow-up. In fact, we speculated that even though the matched baseline characteristics were similar between the two groups, the impact of the cardiovascular risk factors might have a more deep impact on patients who have previously undergone open-heart surgery.

### Limitations

This report is affected by several limitations. First, it is a single-center observational analysis. Secondly, the preoperative decision to perform a surgical or percutaneous approach may resent several inter-operator biases. Another limitation is related to our practice in the management of the discharge of the surgical or percutaneous transfemoral TAVR patient; as it is an internal routine and there is currently a lack of evidence-based guidelines, a final conclusion on the benefit of either the rehabilitation or home discharge can not be reached. Finally, most of the experience was along the balloon-expandable platform, making it hard to drive comparison with other platform sheaths.

## 5. Conclusions

The preoperative selection of the patients based on MSCT is mandatory to improve the vascular and general outcome of the TAVR procedures. The percutaneous approach in the selected population drives a fast in-hospital length of stay and home-based recovery. In our series, it is also linked to better survival rates. In the new parading of tailoring the management of the structural heart disease patient, we believe that handling both techniques (i.e., surgical and percutaneous) could be of worth in the best-option treatment, given their feasibility and good results when properly chosen.

## Figures and Tables

**Table 1:** Baseline characteristics of the unmatched groups (surgical cut-down versus percutaneous) transcatheter aortic valve replacement (TAVR).

	Unmatched		
Variables	Surgical Cut-Down	Percutaneous	*p*-Value
Total Population (*n*)	219	225	
Age, Median (q1–q3)	81 (77–85)	83 (79–86)	<0.01
Male, *n* (%)	105 (47.9)	130 (57.8)	0.04
Female, *n* (%)	114 (52.1)	95 (42.2)	
BMI, Median (q1–q3), kg/m^2^	25.1 (22.2–28.7)	25.1 (23.4–28.0)	0.37
Hypertension, *n* (%)	166 (76.2)	180 (80.0)	0.36
Diabetes, *n* (%)	55 (25.2)	52 (23.1)	0.66
COPD	13 (6)	11 (5)	0.33
Peripheral Vascular Disease, *n* (%)	82 (37.6)	55 (24.4)	<0.01
Creatinin, Median (q1–q3), mg/dL	1.00 (0.81–1.26)	1.02 (0.81–1.29)	0.35
Hb, Mean (SD), (g/dL)	12.4 (1.7)	12.6 (1.6)	0.34
EuroSCORE II log, Median (q1–q3)	4.13 (2.52–6.75)	3.77 (2.33–5.22)	0.21
Atrial Fibrillation, *n* (%)	36 (16.7)	34 (15.3)	0.70
Mean Aortic Gradient, Median (q1–q3), mmHg	46 (39–55)	43 (35–52)	0.02
EF, Median (q1–q3)	61 (53–67)	59 (50–66)	0.05
PAPs, median (q1–q3), mmHg	35 (31–42)	35 (31–42)	0.22

Legend: BMI = body mass index; COPD = chronic obstructive pulmonary disease; Hb = hemoglobin; EF = ejection fraction; PAPs = pulmonary artery pressures; q1 = first quartile; q3 = third quartile.

**Table 2:** Baseline characteristics of the matched groups (surgical cut-down versus percutaneous) TAVR.

	Unmatched		
Variables	Group 1 Surgical Cut-Down	Group 2 Percutaneous	*p*-Value
Total Population (*n*)	119	225	
Age, Median (q1–q3)	83 (78–85)	83 (79–86)	0.45
Male, *n* (%)	67 (56.3)	130 (57.8)	0.79
Female, *n* (%)	52 (43.7)	95 (42.2)	
BMI, Median (q1–q3), kg/m2	24.7 (22.3–27.5)	25.1 (23.4–28.0)	0.05
Hypertension, *n* (%)	89 (74.8)	180 (80.0)	0.27
Diabetes, *n* (%)	27 (22.7)	52 (23.1)	0.93
COPD	7 (6)	11 (5)	0.21
Peripheral Vascular Disease, *n* (%)	37 (31.1)	55 (24.4)	0.19
Creatinin, Median (q1–q3), mg/dL	1.00 (0.82–1.32)	1.02 (0.81–1.29)	0.81
Hb, Mean (SD), (g/dL)	12.5 (1.6)	12.6 (1.6)	0.57
EuroSCORE II log, Median (q1–q3)	4.09 (2.61–7.29)	3.77 (2.33–5.22)	0.22
Atrial Fibrillation, *n* (%)	22 (18.6)	34 (15.3)	0.44
Mean Aortic Gradient, Median (q1–q3), mmHg	45 (38–54)	43 (35–52)	0.30
EF, Median (q1–q3)	61 (48–67)	59 (50–66)	0.35
PAPs, Median (q1–q3), mmHg	35 (31–42)	35 (31–42)	0.37

Legend: BMI = body mass index; COPD = chronic obstructive pulmonary disease; Hb = hemoglobin; EF = ejection fraction; PAPs = pulmonary artery pressures; q1 = first quartile; q3 = third quartile.

**Table 3:** Cardiovascular baseline characteristics of the matched populations.

	Matched Populations		
Variables	Group 1	Group 2	*p*-Value
History of Coronaropathy, *n* (%)	45 (37.8)	92 (40.9)	0.58
Previous CABG o PCI, *n* (%)	19 (16.0)	50 (22.2)	0.17
Previous Cardiac Surgery (%)	31 (26.1)	39 (17.3)	0.07
Previous SAVR, *n* (%)	8 (7.0)	0 (0.0)	<0.01
Severe Peripheral Vascular Disease, *n* (%)	37 (31.1)	55 (24.4)	0.19
EF, Median (q1–q3)	61 (48–67)	59 (50–66)	0.35

Legend: CABG = coronary artery bypass grafting; PCI = percutaneous coronary interventions; SAVR = surgical aortic valve replacement; EF = ejection fraction.

**Table 4:** Procedural features of the matched populations.

Variable	Group 1	Group 2	*p*-Value
Type of Anesthesia			
Deep Sedation, *n* (%)	119 (100.0)	224 (99.5)	1.0
Local Anesthesia + Mild Sedation, *n* (%)	0 (0.0)	1 (0.5)	
Type of TAV			
Self-Expanding TAV, *n* (%)	5 (4.2)	12 (5.3)	0.80
Balloon-Expandable TAV, *n* (%)	114 (95.8)	213 (94.7)	
TAV’s Diameter			
20 mm, *n* (%)	3 (2.5)	1 (0.5)	0.17
23 mm, *n* (%)	52 (43.7)	87 (38.7)	
26 mm, *n* (%)	47 (39.5)	109 (48.4)	
29 mm, *n* (%)	17 (14.3)	28 (12.4)	
Femoral Sheaths			
14F eSheath *n* (%)	100 (84)	191 (84.9)	0.35
14F EnVeo R InLine Sheath *n* (%)	5 (4.2)	12 (5.3)	
16F eSheath *n* (%)	14 (11.8)	22 (9.8)	

Legend: TAV = transcatheter aortic valve.

**Table 5:** Procedural results (Valve Academic Research Consortium-2 definitions) of the matched populations.

Outcome	Group 1	Group 2	*p*-Value
Intraprocedural Death, *n* (%)	1 (0.8)	5 (2.2)	0.67
Cardiac Arrest, *n* (%)	1 (0.8)	4 (1.8)	0.66
Cardiovascular Mortality, *n* (%)	1 (0.8)	4 (1.8)	0.66
More than Mild PVL, *n* (%)	7 (6.0)	19 (8.7)	0.09
Device Embolization, *n* (%)	0 (0)	0 (0)	0.00
Need for CPB/ECMO, *n* (%)	1 (0.8)	1 (0.4)	1.00
Conversion to Sternotomy, *n* (%)	1 (0.8)	1 (0.4)	1.00
Device Success, *n* (%)	116 (98.3)	219 (97.3)	0.72
Minor Vascular Complications, *n* (%)	6 (5.1)	10 (4.5)	0.97
Major Vascular Complications, *n* (%)	3 (2.6)	6 (2.7)	1.00
Coronary Occlusion, *n* (%)	0 (0)	1 (0.4)	1.00
New Onset AF, *n* (%)	6 (5.1)	13 (5.8)	0.79
AMI, *n* (%)	1 (0.8)	0 (0)	0.34
Minor Neurological Events, *n* (%)	1 (0.85)	2 (0.9)	0.48
Major Neurological Events, *n* (%)	0 (0.0)	5 (2.2)	0.38
Major Bleedings, *n* (%)	9 (7.7)	22 (9.9)	0.78
Minor Bleedings, *n* (%)	3 (2.6)	3 (1.3)	0.44
PPI, *n* (%)	3 (5.6)	12 (5.4)	0.28
Temporary Postoperative CVVH, *n* (%)	1 (0.8)	2 (0.9)	1.00

Legend: PVL = paravalvular leak; CPB = cardio-pulmonary bypass; ECMO = extracorporeal membrane oxygenation; AF = atrial fibrillation; AMI = acute myocardial infarction; PPI = permanent pacemaker implantation; CVVH = continuous veno-venous hemofiltration.

**Table 6:** Outcomes and follow-up of the matched populations.

Outcomes	Group 1	Group 2	*p*-Value
Length of Stay, Median (q1–q3), days	7 (5–9)	5 (4–7)	<0.01
Discharged Home, *n* (%)	18 (15.5)	194 (88.2)	<0.01
Rehabilitation, *n* (%)	98 (84.5)	26 (11.8)	<0.01
Follow-Up Mortality, *n* (%)	37 (31.6)	48 (21.8)	0.05
Follow-Up Cardiovascular Mortality al, *n* (%)	11 (9.4)	22 (10.1)	0.85
Median Follow-Up (q1–q3), Days	949 (624–1434)	1039 (703–1553)	0.27
NYHA 1 at Follow-Up, *n* (%)	94 (83.9)	184 (86.4)	0.83
NYHA 2 at Follow-Up, *n* (%)	16 (14.3)	26 (12.2)	
NYHA 3 at Follow-Up, *n* (%)	2 (1.8)	3 (1.4)	
Cardiovascular Rehospitalization, *n* (%)	7 (6.3)	22 (10.3)	0.22

Legend: NYHA = New York Heart Association; q1 = first quartile; q3 = third quartile.

## Data Availability

All the data concerning this work are available from the corresponding author upon reasonable request.
